# Structural damage reduction in protected gold clusters by electron diffraction methods

**DOI:** 10.1186/s40679-016-0026-x

**Published:** 2016-09-26

**Authors:** Eduardo Ortega, Arturo Ponce, Ulises Santiago, Diego Alducin, Alfredo Benitez-Lara, Germán Plascencia-Villa, Miguel José-Yacamán

**Affiliations:** Department of Physics and Astronomy, The University of Texas at San Antonio, One UTSA Circle, San Antonio, TX 78249 USA

**Keywords:** Scanning transmission electron microscopy, Nanobeam electron diffraction, Thiolate-protected gold clusters, Damage reduction

## Abstract

The present work explores electron diffraction methods for studying the structure of metallic clusters stabilized with thiol groups, which are susceptible to structural damage caused by electron beam irradiation. There is a compromise between the electron dose used and the size of the clusters since they have small interaction volume with electrons and as a consequence weak reflections in the diffraction patterns. The common approach of recording individual clusters using nanobeam diffraction has the problem of an increased current density. Dosage can be reduced with the use of a smaller condenser aperture and a higher condenser lens excitation, but even with those set ups collection times tend to be high. For that reason, the methods reported herein collects in a faster way diffraction patterns through the scanning across the clusters under nanobeam diffraction mode. In this way, we are able to collect a map of diffraction patterns, in areas with dispersed clusters, with short exposure times (milliseconds) using a high sensitive CMOS camera. When these maps are compared with their theoretical counterparts, oscillations of the clusters can be observed. The stability of the patterns acquired demonstrates that our methods provide a systematic and precise way to unveil the structure of atomic clusters without extensive detrimental damage of their crystallinity.

**Electronic supplementary material:**

The online version of this article (doi:10.1186/s40679-016-0026-x) contains supplementary material, which is available to authorized users.

## Background

In electron diffraction, it has been of great interest the quantification of intensities for the refinement of structures through electron crystallography methods [1, 2]. However, two limitations need to be overcome; first is related to the sensitivity of the sensors and second is related to the dynamical effects which enable the presence of forbidden reflections [3–5]. In the case of instrumentation, great effort has been made to reduce dynamical effects, caused by multiple scattering events, and to avoid the saturation and blooming effects, produced by the high intensity of the transmitted beam, on its nearest reflections [6]. The reduction of the dynamical effects have been demonstrated by precession electron diffraction [7] and the blooming effect problem has been eliminated using CMOS sensors with high sensitivity even capable of detecting single electron events [8, 9]. Concerning the stability of the sample, Egerton et al. has previously shown how electron beam radiation can cause different types of damage in a specimen [10, 11]. In order to reduce this radiation effects, low electron dose (number of electrons per area) techniques can be implemented. However, a critical compromise between damage and the availability of detecting electron for low dose imaging needs to be taken into account as reducing the incident beam current prolongs the time required to record an image or spectrum, increasing the likelihood of the specimen or high-voltage drift; nevertheless, it lessens the risk of thermal decomposition or electrostatic charging influence. Furthermore, mass loss (directly related to structural damage) can be reduced by cooling the specimen using special cryo-holders. A combination of all these protocols are able to reduce the dose rate as well as the accumulated dose giving the sample more time to dissipate and diminish charge effects [12].

The analysis of metallic thiol-protected clusters is affected by two important mechanisms of radiation damage: radiolysis (for the mercaptans) and knock on (for the metallic core). For organic solids, the damage decreases with higher accelerating voltages, lower beam currents and reduced exposition times [13]. Nevertheless, changes to the sample caused by irradiation are not only restricted to its organic ligands. As an example, the splitting of metal clusters from larger crystals has been demonstrated using an STEM/NBD setup with a probe current density around ∼6 × 10^8^  ${\bar{\text{e}}}$ Å^−2^ s^−1^ [14]. The phenomenon can be explained by the beam-induced surface mobility of metal atoms on the nanoparticle, as in some metals, e.g., gold, this threshold energy for bulk displacement can be as low as 34 eV [15]. This illustrate how the 200 kV electron energy in normal transmission electron microscopy (TEM) studies is capable of rotate and coalesce nanoparticles of heavy atoms (e.g., Pt, Au, Hg) whose knock on energies are above this range. Radiation-enhanced diffusion should consequently be considered as a driving force leading to the reconfiguration of surface atoms [16]. Verification of the atomic structure of sensitive clusters is still developing as researchers have been incorporating the capabilities of STEM in the electron diffraction analysis. Among others, low dose investigation has been realized on the power spectral density of STEM images from strontium titanate, where changing only operational scanning parameters, doses of 450, 30 and 15 ${\bar{\text{e}}}$ Å^−2^ has been recorded [17, 18].

In this work, we present a method toward the structural analysis of protected gold clusters using rapid nanobeam diffraction (NBD) in scanning transmission electron microscopy (STEM), we use as an example the Au_102_(p-MBA)_44_ nanocluster [19, 20]; however, we have carried out experiments to demonstrate structural changes in other metallic clusters such as the Au_144_(SCH_2_CH_2_Ph)_60_ [21]. The structure has been compared between experimental and simulated electron diffraction patterns. Conventional TEM using selected area electron diffraction (SAED) has also been used to evaluate the changes in the structure of the nanoclusters. In that way, STEM/NBD helps to reduce damage in sensitive materials, based on the short acquisition of excellent coherent diffraction patterns for bulk and individual nanoparticles [22, 23].

## Methods

### Sample preparation

**Fig. 1:**
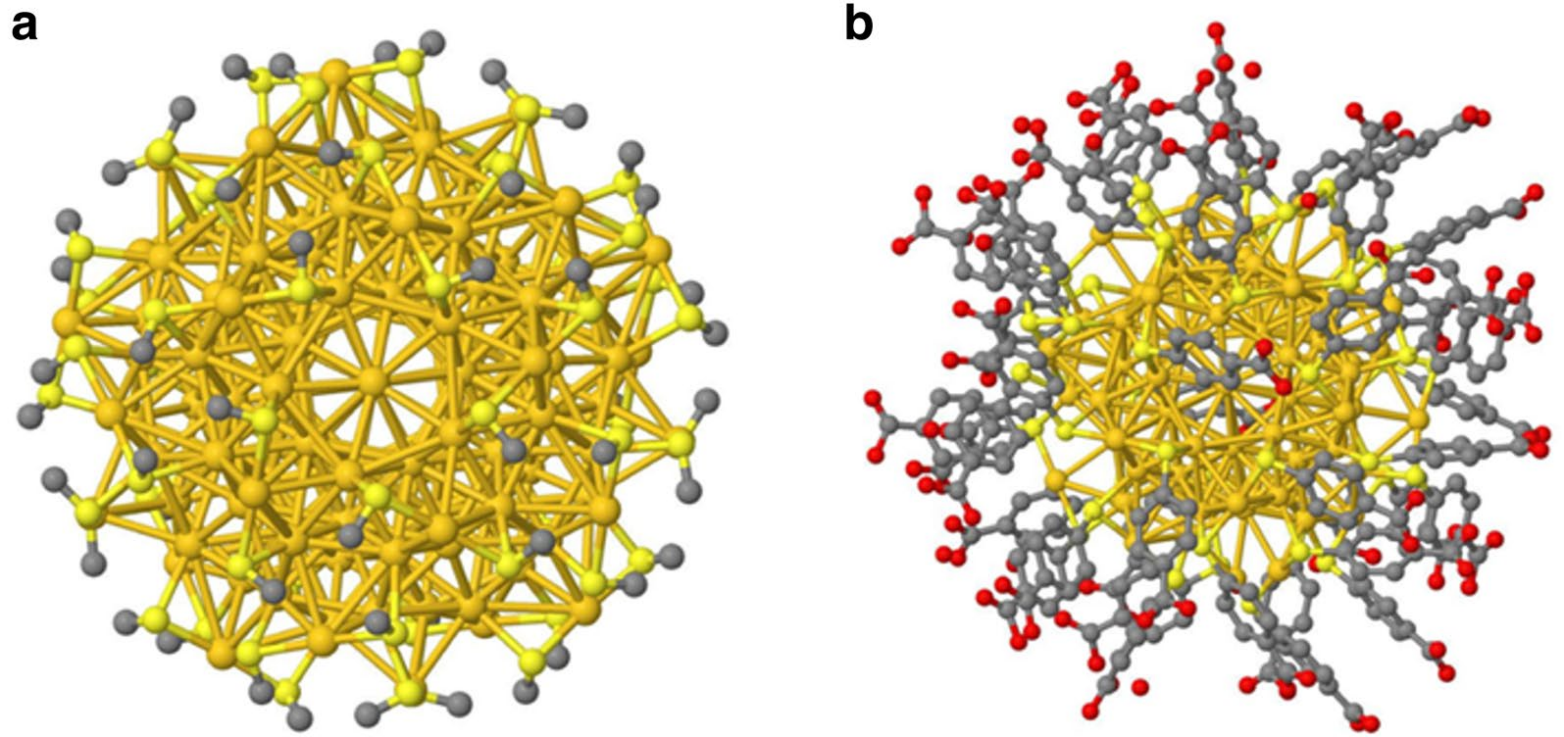
Theoretical structures used for simulation. **a** Au_144_(SR)_60_ [21] and **b** Au_102_(MBA)_44_ [20] oriented along the fivefold direction

The gold clusters (Au_102_-pMBA_44_ and Au_144_(SCH_2_CH_2_Ph)_60_) were produced by the two-phase transfer method [19, 20], their expected structures are shown in Fig. 1, both nanoparticles have been optimized by first-principles density functional theory (DFT) calculations [19, 21]. Quality and size distribution of Au clusters were confirmed by polyacrylamide gel electrophoresis and electrospray ionization mass spectrometry (ESI–MS) using a Bruker micro-TOF instrument. The stock Au clusters were diluted 100-fold with ddH_2_O, then 3–4 drops (20 µl) were loaded on a holey carbon film-coated grids and air dried at room temperature for at least 2 h.

### Scanning nanobeam electron diffraction

**Fig. 2:**
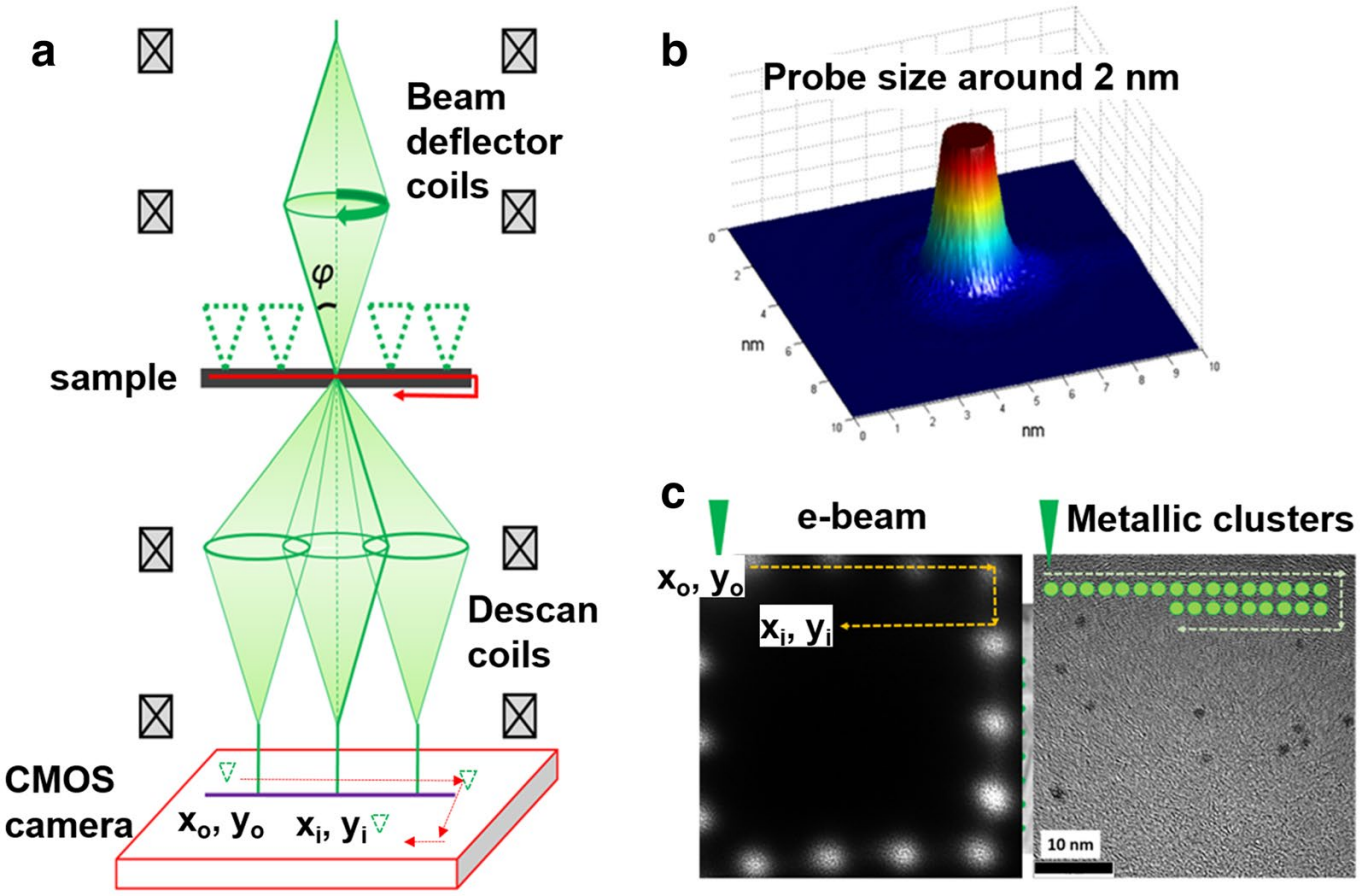
Experimental setup of the S-NBD technique. **a** Schematic representation of the transmission electron microscope and the synchronized collection of electron diffraction patterns using a high sensitive CMOS camera. **b** Beam size used for the scanning diffraction method and **c** example of a nanobeam scanned area and its perimeter recorded at the CMOS camera

The electron diffraction was performed in a JEOL 2010F microscope operated at 200 kV. The scanning of the electron beam is possible using a precession electron diffraction-assisted automated crystal orientation mapping (PED ACOM-TEM) unit attached to the microscope [24]. In the ACOM-TEM technique, the electron beam is scanned across the sample and collects the electron diffraction patterns using an ultrafast charge-coupled device (CCD) camera attached to the viewing screen of the microscope. The CCD camera allows us to obtain an image of the scanned area. However, the CCD camera is not sensitive enough to collect the patterns due to the small volume of the clusters which produce weak reflections. In this way, we have synchronized the scanning and the acquisition of the patterns with an ultrafast TVIPS 16-mega pixel F416 CMOS camera with a dynamic range (max./noise) of 10,000:1. This CMOS camera eliminates streaking problem for high intensity reflections and transmitted beam in electron diffraction suffered in regular CCD cameras. A schematic representation of the experimental setup is illustrated in Fig. 2a, the probe size is about 2 nm as show in the surface plot image of the Fig. 2b. In scanning electron diffraction, the electron beam is tilted and subsequently de-scanned in a complementary way with the image shift coils, so that the diffraction pattern appears as a stationary spot pattern and the scanning is carried out line-by-line of the field of view selected using the external CCD camera in front of the screen of the microscope as show in Fig. 2c. The patterns are recorded with the CMOS camera in video mode which is capable to register patterns every 0.1 s by means of a line-by-line sweep and saved in individual images which are subsequently processed and compared with simulated patterns of the theoretical structure. The scanning NBD allows the collection of the patterns, before any irreversible change in the cluster structure may arise following electron beam irradiation of a static beam in conventional TEM.

## Results and discussion

### Electron diffraction under continuous irradiation

Brightness is directly related to the electron current density per unit solid angle of the source. The current density can be measured from the fluorescence screen to convert it to the dose rate on the specimen. However, to determine the dose rate, the magnification factor, which is also proportional to the radius of the viewing screen, needs to be taken into account. In the microscope, this magnification is referred to the film plane; although there are no films in these days, the magnifications still use that plane as a reference. Hence, the magnification of the images recorded using a camera corresponds to around 80 % of that on the film plane. Therefore, we can relate the current density on the screen to that on the specimen by the following formula:

1 $$\sigma = \rho \times C \times (0.8{M})^{2}$$

where σ is the number of electrons per area (${\bar{\text{e}}}$ Å^−2^), ρ is the current density measured on the screen viewing (pA cm^−2^), *M* is the magnification on the screen, and C (∼6.25 × 10^−10^${\bar{\text{e}}}\,{\text{cm}}^{2} {\text{pA}}^{ - 1}$ Å^−2^) is a proportionality constant to relate the current per cm^2^ to electrons per Å^2^, units need to be converted properly considering that the dose or dose rate is calculated within an specific exposure time in which the shutter is open to irradiate the sample.

**Fig. 3:**
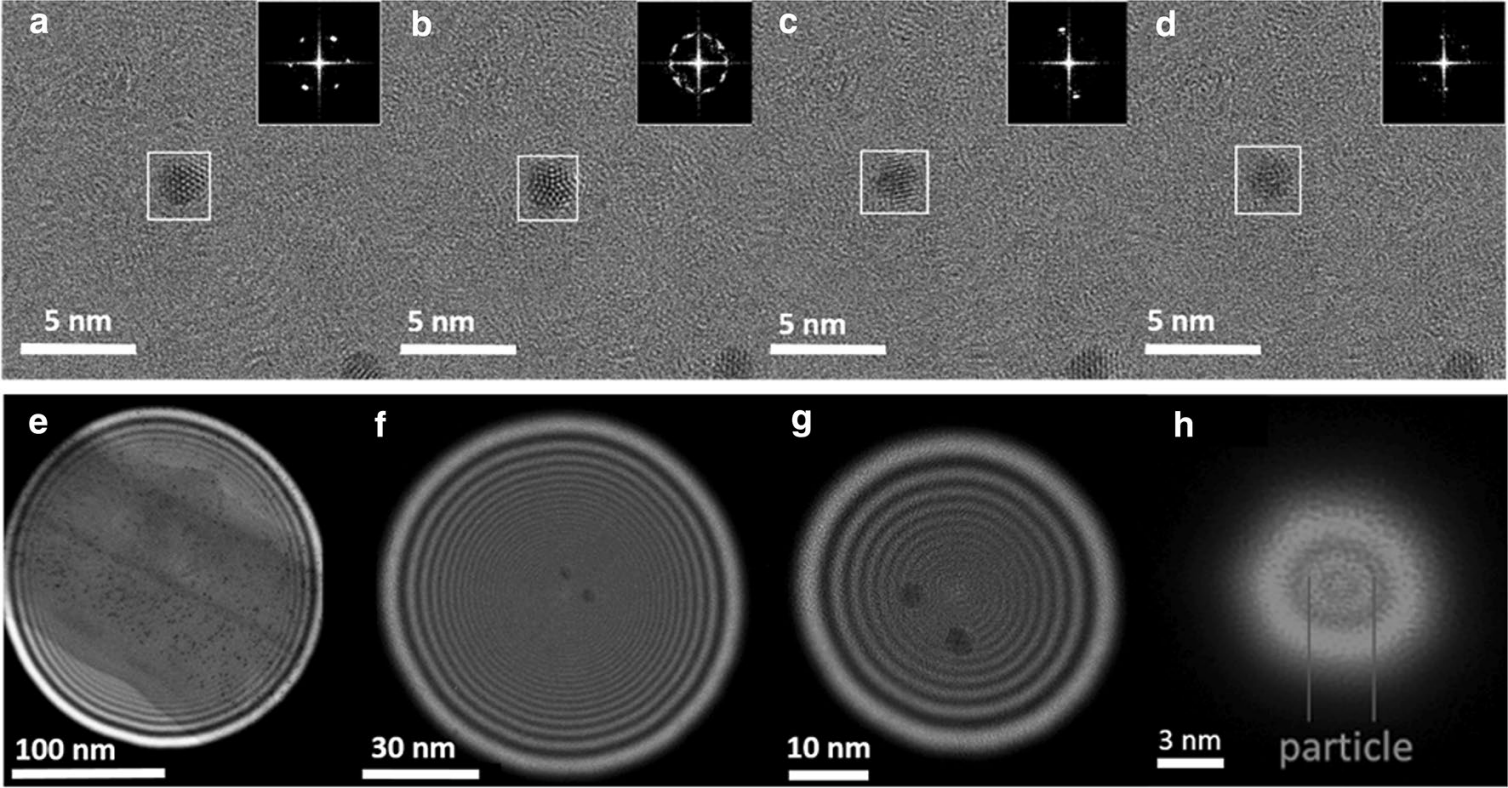
Effects and variations of electron dosage in metallic nanoparticles. Frame shot sequence of Au_144_(SCH_2_CH_2_Ph)_60_ on amorphous carbon. The *insets* show the FFT for the framed region, the structure of the particle is modified by the irradiation: **a** fcc-like orientation, **b** fivefold orientation, **c** and **d** other two different orientations. TEM-Nanobeam-diffraction irradiated areas at different magnifications, the dose rates calculated within the screen of the microscope are: **e** 15 ${\bar{\text{e}}}$ Å^−2^ s^−1^, **f** 80 ${\bar{\text{e}}}$ Å^−2^ s^−1^, **g** 400 ${\bar{\text{e}}}$ Å^−2^ s^−1^ and **h** 13,500 ${\bar{\text{e}}}$ Å^−2^ s^−1^

Conventional high-resolution transmission electron microscopy (HRTEM) and selected area electron diffraction (SAED) are typically acquired with a large condenser aperture (30–40 μm) which yields density currents, recorded on the phosphorous plate, of around 100 pA cm^−2^. If we consider radiolysis as the primary radiation damage in the studied clusters, then for a HRTEM image taken at M = 500 K the electron dose exerted on the sample will be around 12,500 ${\bar{\text{e}}}$Å^−2^, this dose rate is capable of producing changes in the structure of the metallic clusters because the thiol groups are more sensitive and are located in the surface of the metallic core atoms. The images shown in Fig. 3a–d indicate the structural transformations from an individual Au_144_(SR)_60_ cluster, the structure has been compared with the relaxed structure calculated in Ref. [20] (see the full video in Additional file 1). Experimental HRTEM observations of gold nanoparticles show the occurrence of structural instabilities, such as quasi melting as reported in the literature [25], this process is produced by existence of multiple structural configurations separated by low energy barriers. Hence, to diminish the radiolysis effect, the nanoprobe conditions are set up in the microscope with a small condenser lens aperture (5 μm) and a large demagnification of the condenser lens in the JEOL 2010F microscope. Under nanoprobe mode a sample can be irradiated in a quasi-parallel illumination reducing the current density in about two orders of magnitudes. The images shown in Fig. 3e–h represent a sequence of the irradiated area in Au_144_(SR)_60_ clusters. In these series, the current density increases as the irradiated area decreases. At low magnifications, the structure transformations are drastically reduced but are still present after few seconds in which the beam is positioned in the cluster as we demonstrated in a previous article using nanobeam diffraction in STEM mode [21]. In order to register proper diffraction patterns with a reduced noise-signal ratio, the patterns need to be registered with the minimum probe size, which increases the electron dose in almost three orders of magnitude as depicted when comparing Fig. 3e, h. To protect these clusters from radiolysis effects the approach then relies in a reduction of the acquisition time using a fast scanning and fast detection experimental set ups.

### Scanning nanobeam electron diffraction

**Fig. 4:**
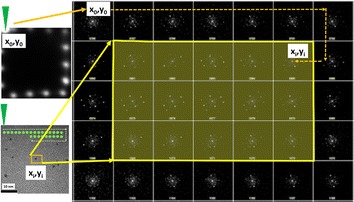
Data acquisition on a S-NBD technique. Set of electron diffraction patterns acquired on a scanned area at 500 K× in a JEOL 2010F, taken with a scanning time of 100 ms per pattern the estimated radiation dose, recorded within the screen of the microscope, is ∼1350 ${\bar{\text{e}}}$ Å^−2^

The acquisition of the scanning nanobeam diffraction patterns was performed with a probe current of 2 pA cm^−2^ and a probe size of 2 nm to scan ~1.6 nm Au_102_(MBA)_44_ cluster. As shown in Fig. 3h the patterns have been registered with an estimated dose of 13,500 ${\bar{\text{e}}}$ Å^−1^s^−1^. This dose rate can then be reduced adjusting the acquisition/interaction time of the probe with the nanoparticle during the scanning procedure. In this setup, each pattern is collected every 100 milliseconds yielding a dose of approximately 1350  ${\bar{\text{e}}}$ Å^−2^. Due to the probe size and the scanning steps, the electron beam interacts with the cluster more than one time, causing oscillations that can be detected in the adjacent patterns of one cluster. The patterns shown in Fig. 4 represent a fraction of the whole area scanned and registered in the CMOS camera. The patterns highlighted within the yellow square correspond to frames surrounding an individual cluster. The indexing of these patterns have been analyzed using the xyz cluster coordinates from the relaxed structure of the Au_102_(p-MBA)_44_ cluster determined by X-ray diffraction and optimized by density functional theory (DFT) [21]. The simulated diffraction patterns were indexed using the module “Nanodiffraction” in the java electron microscopy simulations software package [26]. Image processing has been made in order to enhance the features already present in the images, those filters and parameter were implemented for all images acquired.

**Fig. 5:**
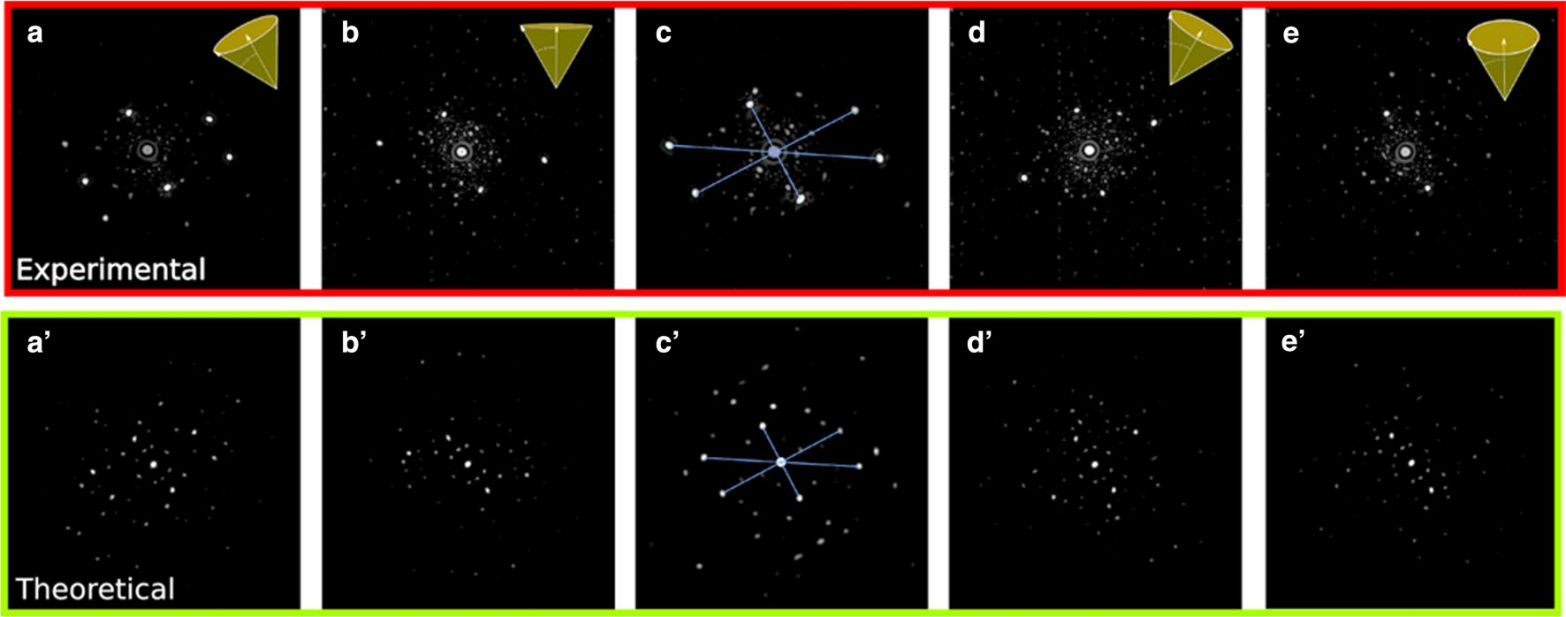
Set of experimental STEM/NBD and simulated patterns extracted from the Au_102_(p-MBA)_44_ cluster. Given an arbitrary orientation of the nanoparticle (**c**), a conical oscillation of the cluster is observed from the surrounding diffraction patterns (**a**, **b**, **d**, **e**), these one degree variations correspond to a “*left*, *straight*, *right* and *back*” tilting

Using this fast scanning electron diffraction method we are able to record and assess the structure of the Au_102_(p-MBA)_44_ cluster with an outstanding precision. Due both, the beam probe size and the scan spacing, a single particle can interact with the electron beam several times, this diffraction patterns possess certain similarities but are not equal as depicted on Fig. 5. From this information, we conclude that the particle is oriented almost in the same direction during scanning, i.e., the beam-particle interaction is such that it does not significantly disturb the state (position) of the particle. The simulation procedure for obtain this small angle variations is based on creating a library of diffraction patterns from a given arbitrary position of the Au_102_(p-MBA)_44_ structure, that is both azimuth and zenith angle rotated at intervals of one degree. Therefore, each randomly deposited cluster, will produce an experimental pattern that match our library. The map of patterns in the area within the cluster and its surrounding shows a good agreement when is indexed with the simulated patterns of the cluster disoriented one degree. Under the conditions described even after multiple recording events the clusters preserve their structure. The opposite effect, has been observed in a previous work where a long exposure time of the nanobeam diffraction patterns completely transformed the structure of metallic clusters when the electron beam remains static for a few seconds over the clusters [27].

The rapid collection of the diffraction patterns presented in the current work is an efficient method for preventing damage in sensitive small clusters without deterioration of the structure and the loss of the thiol groups which stabilize the metallic structure.

## Conclusion

In summary, we demonstrated a systematic scanning nanobeam diffraction method which relies on a fast acquisition of electron diffraction patterns using a high sensitive CMOS detector. The method reduces the deterioration of the structure of the sensitive protected metallic clusters and makes possible the determination of their structure, comparing the experimental data with the simulated patterns of the optimized structures. A nominal electron dose of 1350 ${\bar{\text{e}}}$ Å^−2^ per recorded pattern is exerted during the data acquisition which can be compared with their counterpart doses in conventional selected area electron diffraction of about 1250 ${\bar{\text{e}}}$ Å^−2^. Although the rate doses of the two methods are almost the same, the advantage of the NBD setup relies on the acquisition of specific local media as opposed with conventional SAED where we collect the superimpose information of all the enclosed area (other crystallites, nanoparticles, amorphous carbon, etc.) which makes possible even differentiate small angle rotations produced by the interaction of the clusters with the electron probe. This methodology can be used for other small nanoparticles or sensitive specimens in which a precision and high-resolution are required to avoid excessive radiation damage mechanisms.

## Additional file

10.1186/s40679-016-0026-x Continuous structural transformation of a gold cluster due to electron beam irradiation.
